# Quercetin Improves Pulmonary Function and Prevents Emphysema Caused by Exposure to Cigarette Smoke in Male Mice

**DOI:** 10.3390/antiox11020181

**Published:** 2022-01-18

**Authors:** Natália Pereira da Silva Araújo, Natália Alves de Matos, Michel Oliveira, Ana Beatriz Farias de Souza, Thalles de Freitas Castro, Pedro Alves Machado-Júnior, Débora Maria Soares de Souza, André Talvani, Sílvia Dantas Cangussú, Rodrigo Cunha Alvim de Menezes, Frank Silva Bezerra

**Affiliations:** 1 Laboratory of Experimental Pathophysiology, Department of Biological Sciences and Center of Research in Biological Sciences, Federal University of Ouro Preto (UFOP), Ouro Preto 35400-000, Brazil; natalia.araujo@aluno.ufop.edu.br (N.P.d.S.A.); natalia.matos@ufop.edu.br (N.A.d.M.); michel.oliveira@aluno.ufop.edu.br (M.O.); ana.farias@aluno.ufop.edu.br (A.B.F.d.S.); thalles.castro@aluno.ufop.edu.br (T.d.F.C.); pedro.junior@aluno.ufop.edu.br (P.A.M.-J.); cangussu@ufop.edu.br (S.D.C.); 2 Laboratory of Immunobiology of Inflammation, Department of Biological Sciences, Institute of Exact and Biological Sciences, Federal University of Ouro Preto (UFOP), Ouro Preto 35400-000, Brazil; debora.souza@aluno.ufop.edu.br (D.M.S.d.S.); talvani@ufop.edu.br (A.T.); 3 Laboratory of Cardiovascular Physiology, Department of Biological Sciences and Center of Research in Biological Sciences, Federal University of Ouro Preto (UFOP), Ouro Preto 35400-000, Brazil; rodrigo.menezes@ufop.edu.br

**Keywords:** quercetin, cigarette smoke, oxidative stress, COPD, emphysema

## Abstract

Chronic obstructive pulmonary disease (COPD) is the major cause of morbidity and mortality worldwide, and cigarette smoke is a key factor in the development of COPD. Thus, the development of effective therapies to prevent the advancement of COPD has become increasingly essential. We hypothesized that quercetin protects lungs in mice exposed to long-term cigarette smoke. Thirty-five C57BL/6 mice were exposed to cigarette smoke (12 cigarettes per day) for 60 days and pretreated with 10 mg/kg/day of quercetin via orogastric gavage. After the experimental protocol, the animals were euthanized and samples were collected for histopathological, antioxidant defense, oxidative stress and inflammatory analysis. The animals exposed to cigarette smoke showed an increase in respiratory rate and hematological parameters, cell influx into the airways, oxidative damage and inflammatory mediators, besides presenting with alterations in the pulmonary histoarchitecture. The animals receiving 10 mg/kg/day of quercetin that were exposed to cigarette smoke presented a reduction in cellular influx, less oxidative damage, reduction in cytokine levels, improvement in the histological pattern and improvement in pulmonary emphysema compared to the group that was only exposed to cigarette smoke. These results suggest that quercetin may be an agent in preventing pulmonary emphysema induced by cigarette smoke.

## 1. Introduction

Chronic obstructive pulmonary disease (COPD) is the third leading cause of death worldwide, which caused 3 million deaths in 2019 [1]. This disease can be defined as a syndrome characterized by constant respiratory symptoms, progressive airflow obstruction and limitation [2,3]. Tobacco smoking is the key environmental risk factor for COPD [4,5].

Cigarette smoke contains a wide variety of chemical compounds, which are mostly toxic, including reactive oxygen species (ROS) [6]. In the epithelium airways, these substances increase the production and secretion of cytokines and chemokines leading to the recruitment of immune cells, mainly macrophages and neutrophils. These cells maintain the inflammation process and increase ROS production, which in conjunction with the reactive species present in cigarette smoke can exceed the antioxidant capacity of the body, thus causing redox imbalance [7,8]. This imbalance is a major predisposing factor in the pathogenesis and progression of COPD. Excessive ROS cause direct damage to cellular components, such as proteins, lipids and DNA, and increases the expression of pro-inflammatory mediators, besides increasing the expression and activity of metalloproteinases (MMP) [9,10]. These processes lead to degradation of the extracellular matrix and consequent alveolar destruction [9,10].

Glucocorticoids and bronchodilators compose the main pharmacological therapy used in the COPD treatment, aiming to control respiratory symptoms and prevent exacerbations [11,12]. However, the current treatment does not prevent COPD progression effectively [11,12,13], warranting the investigation of novel treatments in order to prevent the advancement of chronic obstructive pulmonary disease.

Quercetin (3,3′,4′,5,7-pentahydroxy flavone) is a phenolic compound, belonging to the flavonoid family. This is the most abundant compound of the flavanol family and is present in a variety of food or produce, such as onions, apples and broccoli [14]. Quercetin has a high antioxidant activity, which depends on several mechanisms, including its ability to react with reactive species and form less reactive phenoxy radicals [14,15]. Moreover, the flavonoid acts to prevent lipid peroxidation by neutralizing various reactive oxygen species. Furthermore, quercetin inhibits various protein kinases, besides being involved in the activation of the Nrf2 pathway, thus improving the activity of antioxidant enzymes and inhibiting the NF-κB pathway [16,17,18]. In addition, this compound acts in the metabolism of MMP by preventing redox imbalance, lung inflammation and expression of MMP9 and MMP12 [14,19].

Previous data from our laboratory showed that quercetin significantly reduces lung inflammation, histological pattern changes of pulmonary parenchyma and lung function changes in short-term exposure to the cigarette smoke model [20]. In addition, a recent study suggested that supplementation with this flavonoid, in the diet of the animals, decreases the rhinovirus-induced lung inflammation in mice with COPD phenotype [21]. Importantly, a clinical trial with COPD subjects showed quercetin consumption was safe and well tolerated by the subjects [22]. Thus, considering our previous results, we hypothesized that quercetin could prevent cigarette smoke damage to the lung. In order to verify the initial hypothesis in the present study, we aimed to examine the quercetin ability of mitigating the long-term effects of cigarette smoke exposure, specifically on pulmonary redox imbalance, inflammation and tissue damage in C57BL/6 mice.

## 2. Materials and Methods

### 2.1. Animals

Thirty-five male C57BL/6 mice (8–10 weeks old) obtained from the Animal Science Center at the Federal University of Ouro Preto (CCA-UFOP), were housed under controlled conditions (12 h light/dark cycle, 21 ± 2 °C, 50 ± 10% humidity), received food (Nuvilab^®^, Table 1) and water ad libitum. The sample size was determined using Biostat software version 5.3 (Mamiraúa Institute, Tefé, Amazonas, Brazil).

**Figure 1:**
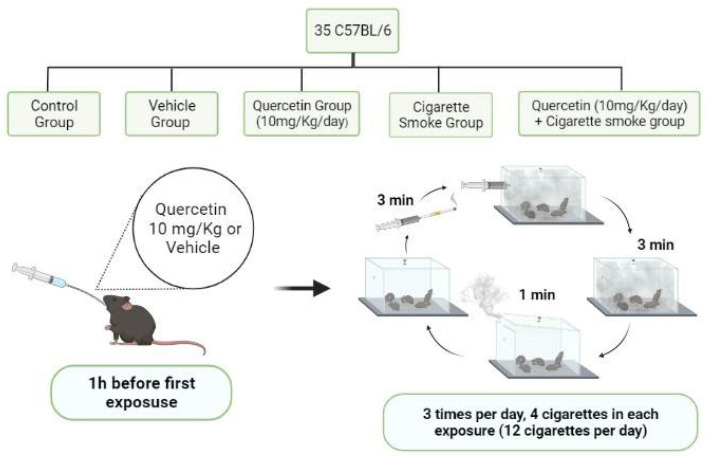
Diagram of the experimental design.

The mice were divided into 5 groups (*n* = 5–7 per group): control group (CG); a vehicle group (VG) quercetin group (QG); cigarette smoke group (CSG); quercetin and cigarette smoke group (QCSG). The animals in the vehicle group received, via orogastric gavage, 200 µL of vehicle solution composed of 50% propylene glycol (LABSYNTH) and 50% saline solution. The animals of QG and QCSG received 10 mg/Kg/day of quercetin diluted in 200 µL of vehicle solution 1 h before the first CS exposure. All procedures performed in this study were approved by the Ethics Committee on Animal Use at the Federal University of Ouro Preto (Protocol number 2015/20, approved on 16 November 2015) (Figure 1).

### 2.2. Cigarette Smoke Exposure Protocol

The animals of CSG and QCSG were exposed for 60 consecutive days to 12 commercial Virginia Full-flavor filtered cigarettes (tar: 10 mg; nicotine: 0.9 mg and carbon monoxide: 10 mg) per day divided into three periods (morning, afternoon and evening) as previously described [23,24]. The animals were initially placed in an inhalation chamber (40 cm length × 30 cm width × 25 cm height) with a capacity of 30 L inside an exhaustion chapel. Each cigarette was coupled to a 60 mL plastic syringe through which the smoke was injected into the inhalation chamber. The time to burn 1 cigarette was 3 min and the smoke generated by the burning was 1 L, representing 3% of the total chamber capacity. The animals were kept in contact with the smoke for 6 min, then the lid of the inhalation chamber was removed for 1 min to allow complete exhaustion of the smoke. After the total time of 7 min, the procedure was repeated for the remaining cigarettes. The animals of CG, VG and QG were subjected to the same conditions, but without exposure to cigarette smoke (Figure 1).

### 2.3. Pulmonary Function

Twenty-four hours after the last exposure, the animals were individually anesthetized with a mixture of ketamine and kilazine (100 mg/kg and 20 mg/kg, respectively) and lung function was collected according to the protocol previously described by Araújo et al. [20]. The following parameters were recorded and analyzed: tidal volume (VT), respiratory rate (RR) and minute ventilation (MV).

### 2.4. Blood Collection

After the collection of lung function parameters, the animals were euthanized with an overdose of ketamine (130 mg/kg) and xylazine (0.3 mg/kg). The thorax of the animals was opened, an incision was made in the third intercostal space and blood was collected by cardiac puncture. The blood was stored in polypropylene tubes containing 15 µL of anticoagulant, and later, using an electronic counting device (Mindray^®^ Bio-Medical Electronics Co. Ltd., Shenzhen, China), the erythrocytes, hematocrit and hemoglobin were evaluated [20].

### 2.5. Collection and Analysis of Bronchoalveolar Lavage Fluid (BALF)

Subsequently, the blood collection, the thorax of each animal was opened and the left main bronchus was clamped, the trachea was cannulated and the right lung was washed with 1.5 mL of saline solution (3 × 500 µL). The bronchoalveolar lavage fluid (BALF) samples were kept on ice until the end of the procedure to avoid cell lysis. A Neubauer chamber was used to determine the total leukocytes, and to perform the differential cell count, 250 μL of each sample was centrifuged in a cytocentrifuge (INBRAS, São Paulo, Brazil). Then, the slides with the cells were stained with a rapid staining kit (Laborclin, Pinhais, Brazil) using an optical microscope; 100 cells were counted per slide [20,25].

### 2.6. Lung Collection

After BALF collection, the right ventricle of each animal was perfused with a saline solution to remove blood cells from the lungs. The right lung was clamped so only the left lung could be perfused with 4% buffered formalin (pH 7.2) at a pressure of 25 cm H_2_O for 2 min via the trachea. The left lung was then removed and immersed in a fixative solution for 48 h, then the material was processed and the slides were stained with hematoxylin and eosin (H&E), Gomori trichrome and Verhoeff for histological analysis. Afterward, the right lung was removed and homogenized in 1 mL of phosphate buffer (pH 7.4) and centrifuged at 15,521× *g* for 10 min. The supernatant was stored at −80 °C for further biochemical analysis.

### 2.7. Immunoenzymatic Assay for Inflammatory Mediators

Interleukins (IL)-17, IL-22, IL-10 and IL-13 were performed in lung homogenates by the enzyme-linked immunosorbent assay (ELISA) method. The assays were performed using commercial kits (PeproTech, Ribeirão Preto, Brazil), and the antibodies and reagents were prepared according to the instructions from the manufacturer. Immunoassays were performed in 96-well plates, in which 100 µL of monoclonal antibodies against the proteins (or peptides) of interest were added. Samples were diluted in PBS containing 0.1% bovine serum albumin (BSA; Sigma-Aldrich, St. Louis, MO, USA). After incubation for 12 h at room temperature, the plates were blocked with 300 µL/well of a PBS solution containing 1% BSA for 1 h at 37 °C. Samples were added in a volume of 25 µL to each well. Intensity reading was performed in an ELISA reader at 490 nm and the quantification obtained as previously described [24].

### 2.8. Biomarkers of Oxidative Stress and Antioxidant Defense

In order to evaluate and measure the activity of antioxidant enzymes and oxidative damage, lung homogenates were used. Superoxide dismutase (SOD) activity was measured according to the Marklund and Marklund method, which is based on the ability of the enzyme to inhibit oxidation of pyrogallol, after the reaction the samples were read using ELISA reader (λ = 570 nm) [26]. The analysis of catalase activity is based on the conversion of hydrogen peroxide (H_2_O_2_) to water and molecular oxygen (O_2_) per minute, was measured by spectrophotometry (λ = 240 nm) as described by Aebi [27]. Glutathione analysis was determined using an assay adapted from a commercial kit (CS0260, Sigma, St. Louis, MO, USA); the method is based on the ability to reduce 5,5′-Dithio-bis-(2-nitrobenzoic) acid to thio-2-nitrobenzoic acid according to the Griffith test [28]. Initially, a serial dilution standard curve was prepared, and based on the curve, total glutathione (GSH + GSSG) and oxidized glutathione (GSSG) were calculated. The concentration of reduced glutathione (GSH) was calculated by subtracting the oxidized glutathione from the total glutathione value, and then the GSH/GSSG ratio was calculated. Myeloperoxidase (MPO) activity was determined in pulmonary homogenate and 96-well plates at 630 nm according to the protocol described by Campos et al. [23].

Oxidative damage was determined based on the lipid peroxidation of thiobarbituric acid reactive substances (TBARS) following the method described by Buege and Aust [29] and measuring the levels of oxidation-modified proteins were determined according to the adapted protocol of Reznick and Packer [30]. Total protein was measured according to the method of Bradford [31].

### 2.9. Stereological and Morphometric Analysis

In order to assess pulmonary histoarchitecture, collagen and elastic fibers slide stained with H&E, Gomori trichrome, or Verhoeff were analyzed. Twenty random fields from histological sections were captured using a light microscope equipped with an Axiocam 105 color digital camera (Carl Zeiss AG, Oberkochen, Germany) coupled with the ZEN Lite image capture software using a 40× and 20× microscopic objectives. The volume density analysis was performed in a test system composed of 16 points and a known test area, to avoid overestimating the number of structures. The test system was coupled to the monitor and 20 fields were analyzed to obtain uniform and proportional lung samples. The number of points (Pp) that reached the analyzed structures was evaluated according to the total number of points in the test system (Pt) as previously described [32,33]. A total area of 1.94 mm^2^ of each slide for H&E-stained slides determined the volume density of the alveolar airspace (Vv [a]) and alveolar septa (Vv [sa]), the volume density of collagen fibers (Vv [col]) in sections stained with Gomori trichrome and the volume density of elastic fibers (Vv [ela]) in sections stained with Verhoeff.

Suitable for assessing the extent of lung injury (emphysema), morphometric analysis was carried out on sections stained with H&E. The mean linear intercept (Lm) was calculated as an indicator of the airspace size [23,34].

### 2.10. Statistical Analysis

The normal distribution of each variable was assessed using the Kolmogorov-Smirnov test. The normally distributed data were analyzed with one-way ANOVA followed by Tukey’s post-test, and the data are expressed as mean ± standard deviation (SD). For nonparametric data, the Kruskal-Wallis test followed by Dunn’s post-test, and the data are expressed as the median, interquartile range (25% and 75%). In both cases, the difference was considered significant when the p value was <0.05. Statistical analyses were performed using Prism v. 5 (Windows 7, GraphPad Software, San Diego, CA, USA).

## 3. Results

### 3.1. Analysis of Pulmonary Function in the Experimental Groups

Exposure to cigarette smoke (CSG) induced an increase in respiratory rate (ANOVA, *p* = 0.0018) when compared to CG, VG and QG (*p* < 0.05). However, quercetin administration (QCSG) promoted a reduction in respiratory rate when compared to animals that were exposed to cigarette smoke (*p* = 0.01). For minute volume and tidal volume, no significant differences were observed between the experimental groups (Table 2).

### 3.2. Hematological Data

The cigarette smoke group showed higher values for red blood cell (ANOVA, *p* < 0.0001), hemoglobin (ANOVA, *p* < 0.0001) and hematocrit (ANOVA, *p* < 0.0001) analyses when compared to the groups exposed to ambient air (CG, VG, QG) (*p* = 0.001). However, quercetin administration promoted a decrease in the percentage of hematocrit when compared to CSG (*p* = 0.01). For erythrocyte and hemoglobin, the levels kept increasing in QCSG when compared with controls (*p* = 0.001) (Table 3).

### 3.3. Analysis of Quercetin Administration and Cigarette Smoke Exposure on Cell Recruitment to BALF

After 60 consecutive days of exposure, cigarette smoke promoted recruitment of inflammatory cells (ANOVA, *p* < 0.0001), such as macrophages (ANOVA, *p* < 0.0001), lymphocytes (Kruskal-Wallis, *p* = 0.0002) and neutrophils (Kruskal-Wallis, *p* < 0.0001) into the airways compared to the groups exposed to ambient air (CG, VG and QG) (*p* < 0.05). We observed that quercetin administration one hour before exposures resulted in lower recruitment of total leukocytes, especially macrophages compared to CSG (*p* = 0.01) (Table 4).

### 3.4. Inflammatory Cytokine Levels in Pulmonary Homogenate

The inflammatory mediators IL-10, IL-13, IL-17 and IL-22 were determined in the lung homogenate to verify the inflammatory response of the animals. The animals exposed to cigarette smoke showed increased levels of IL-10 (ANOVA, *p* = 0.0068), IL-13 (ANOVA, *p* = 0.0008), IL-17 (ANOVA, *p* = 0.0004) and IL-22 (ANOVA, *p* = 0.0204) compared to the control group (*p* < 0.05). Strikingly, quercetin administration resulted in a decrease in the cytokines IL-10, IL-13 and IL-22 compared to CSG (*p* < 0.05). However, IL-17 levels were increased in QCSG compared to CG (*p* > 0.05) (Table 5).

### 3.5. Biomarkers Analysis of Oxidative Damage and Antioxidant Defense

The animals exposed to cigarette smoke (CSG) showed lower SOD (ANOVA, *p* = 0.0064) and catalase (ANOVA, *p* = 0.0195) activities compared to the control group (*p* < 0.05), the pretreatment with quercetin promoted an increase in the activity of both enzymes when compared to CSG (*p* < 0.05). The GSH/GSSG ratio was lower in the CSG (Kruskal-Wallis, *p* = 0.0002) than in the CG, VG and QG (*p* < 0.05), and the administration with quercetin did not have an effect on this reduction. The myeloperoxidase activity (ANOVA, *p* = 0.0017) was higher in animals exposed to cigarette smoke compared to CG, VG and QG (*p* = 0.01); pretreatment with quercetin promoted a decrease in the activity of this enzyme compared to CSG (*p* = 0.01) (Table 6).

Regarding oxidative damage in the lung parenchyma, the levels of carbonylated protein (ANOVA, *p* < 0.0001) and TBARS (ANOVA, *p* = 0.0013) were evaluated. Both damage markers showed higher levels in the group exposed to cigarette smoke compared to the animals exposed to ambient air (CG, VG and QG) (*p* < 0.05). However, pre-treatment with quercetin (QCSG) resulted in lower levels of protein oxidation and lipid peroxidation compared to the group exposed to cigarette smoke (CSG) (*p* < 0.05) (Table 6).

### 3.6. Morphometric Analyses of the Lung Tissue

**Figure 2:**
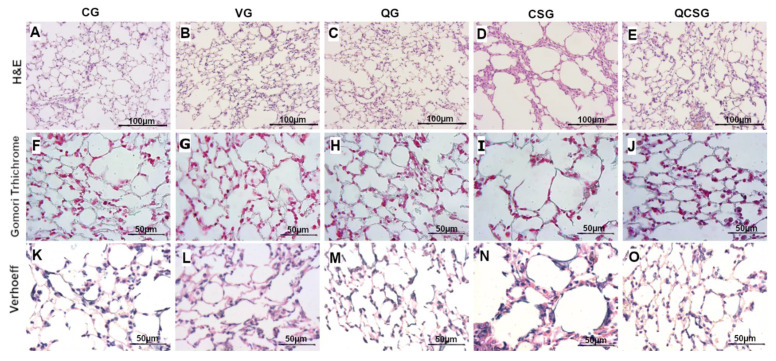
Photomicrographs of lung sections. CG: control group (**A**,**F**,**K**); VG: vehicle group (**B**,**G**,**L**); QG: quercetin group (**C**,**H**,**M**); CSG: cigarette smoke group (**D**,**I**,**N**); QCSG: quercetin and cigarette smoke group (**E**,**J**,**O**). Photomicrographs of lung sections stained with hematoxylin and eosin (**A**–**E**), bar = 100 µm, 200× magnification. Photomicrographs stained in Gomori trichrome (**F**–**J**) and Verhoeff (**K**–**O**), bar = 50 µm, 400× magnification.

Cigarette smoke exposure (CSG) caused alterations in the pulmonary architecture (Figure 2D,I,N)) when compared to CG (Figure 2A,F,K). Importantly, the group pre-treated with quercetin (QCSG) reversed this change (Figure 2E). Collagen and elastic fibers were fragmented and irregular in the CSG (Figure 2I,N) when compared to CG (Figure 2F,K), VG (Figure 2G,L) and QG (Figure 2H,M).

Stereology data confirmed the histological changes. Table 7 shows that the volume densities of the alveolar air space (Vv [a]) (Kruskal-Wallis, *p* < 0.0001) increased in the CSG compared to the CG (*p* < 0.05), and the volume densities of alveolar septa (Vv [sa]) (Kruskal-Wallis, *p* < 0.0001) decreased in CSG compared with the CG (*p* < 0.05). However, quercetin administration promotes a decrease in Vv [a] and an increase in Vv [sa] compared to CSG (*p* = 0.001). In order to evaluate the extent of lung injury, emphysema, caused by prolonged exposure to cigarette smoke, we performed a morphometric analysis of mean linear intercept (Lm), an indicator of airspace enlargement. As can be observed, the Lm CSG (ANOVA, *p* < 0.0001) was elevated in the compared to CG, VG and QG (*p* < 0.0001), and the pretreatment with quercetin (QCSG) was able to improve these effects decreasing the Lm when compared to CSG (*p* < 0.0001). Additionally, animals exposed to cigarette smoke showed a decrease of Vv [col] (Kruskal-Wallis, *p* = 0.0021) and Vv [ela] analysis (Kruskal-Wallis, *p* = 0.0026) in comparison to CG (*p* < 0.05). However, there was no significant difference in mice treated with quercetin (Table 7).

## 4. Discussion

In the present study, we evaluated the effects of quercetin in the morphological changes in lung tissue of mice exposed to cigarette smoke for 60 days with a COPD murine model. We observed quercetin has a protective role reducing the lung injury caused by long-term cigarette smoke exposure. In addition, quercetin reduced lung inflammation and the release of inflammatory and oxidative mediators. These findings show quercetin might be a potential alternative to prevent COPD.

COPD can frequently cause polycythemia, increased erythrocytes and hemoglobin concentration [35]. In the present study, the exposure to cigarette smoke increased erythrocytes, hemoglobin and hematocrit. These changes can be attributed to the fact that cigarette smoke is rich in carbon monoxide and para-benzoquinone, substances able to conjugate with hemoglobin and reduce the oxygen transport capacity causing hypoxia, a condition that also plays an important role in COPD [36]. Hypoxia will stimulate the production of erythropoietin with a consequent increase in the production of red blood cells and hemoglobin, as well as activate the xanthine oxidase pathway resulting in the generation of ROS [20,37]. When the amount of oxygen transported to tissues is insufficient, it causes tachypnea to compensate for the lack of oxygen, a situation commonly found in patients with COPD [38]. Interestingly, quercetin restored the hematocrit levels and respiratory rate in the animals exposed to cigarette smoke, suggesting quercetin could help to maintain an adequate percentage of red blood cells in the blood and lung function improvement in COPD.

Exposure to cigarette smoke also leads to an innate and adaptive immune inflammatory response in the airways and lung tissues [5]. Lung macrophages are the key innate immune cells, acting as phagocyte, destroying pathogens and processing inhaled particles, including cigarette smoke [39]. Moreover, these macrophages start and perpetuate the chronic inflammatory response that supports the progressive nature of COPD [39]. In the present study, cigarette smoke exposure led to greater recruitment of macrophages, lymphocytes and neutrophils, suggesting a greater generation of inflammatory mediators and ROS in CSG. We also found that the pretreatment with quercetin reduced the leukocytes’ influx, particularly macrophages, to BALF in animals exposed to cigarette smoke. These data corroborate a previous study of our group, which showed that natural antioxidants can reduce lung inflammation, total leukocytes and the number of macrophages in BALF caused by cigarette smoke [20,23].

The release of inflammatory mediators, such as cytokines, plays a key role in targeting and perpetuating chronic inflammation in COPD [40]. Several cytokines take part in COPD development, including IL-10 released by macrophages, IL-13 produced by Th2 cells, IL-17 and IL-22 produced by Th17 cells [40,41]. Studies have shown that IL-13, IL-17 and IL-22 are associated with disease severity and exacerbation in COPD, and increase in the airways of patients [42,43,44]. Blocking these interleukins reduced acute exacerbations [42,43,44] and resolved the inflammatory process [45]. In particular, IL-22 is a member of the IL-10 cytokine family [46,47]; it is associated in the airway inflammation and increased in cigarette smoke-induced experimental COPD. IL-22 receptor levels also increased in the lungs of mice with experimental COPD compared to controls, suggesting that this cytokine is a useful biomarker in the diagnosis and/or prognosis of chronic lung diseases [42]. Here, using an established murine model of COPD, we demonstrated that the administration of quercetin restored the IL-10, IL-13 and IL-22 to a level similar to the control group; these data highlighted important roles for these cytokines in this model, and may provide important targets for future investigations to understand the disease progression.

Besides the anti-inflammatory effect, quercetin may also indirectly improve the antioxidant defense by increasing Nrf2-driven antioxidant production and decreasing pro-inflammatory cytokine production [48]. In the nucleus, Nrf2 binds to the antioxidant response element and activates antioxidant genes, such as the superoxide dismutase, glutathione and heme oxygenase 1 genes [48,49]. However, a reduction in Nrf2 can cause redox imbalance, severe lung emphysema, epithelial damage and large sensitivity to oxidative stimuli, including cigarette smoke and particulate matter [50]. In our experimental model, we focused on the development of emphysema, easily detectable by lung morphometry, utilizing a continuous long-term exposure to cigarette smoke, which allows for evaluating the development, progression of pathogenic mechanisms of COPD [51,52]. According to this concept, our study demonstrated that cigarette smoke exposure to the animals resulted in pulmonary emphysema with significant damages in the alveolar structure (increase in Lm and Vv [a] and decrease in Vv [sa]), inflammatory mediators and oxidative changes. Interestingly, our results showed that quercetin treatment decreased markers of oxidative stress evaluated by oxidative damage to macromolecules, such as lipids and proteins, decreased myeloperoxidase activity and increased the activity of antioxidant enzymes. The reduction in oxidative stress, added to the reduction of macrophages and inflammatory mediators, caused by quercetin, protected the alveolar structure (decrease in Vv [a] and increase in Vv [sa]) and avoided hyperdistention (decrease in Lm); however, quercetin did not prevent collagen and elastic fiber changes. Together, this evidence strengthens the hypothesis that oxidative stress is involved in the pathogenesis and progression of COPD [23,53], as redox imbalance caused by prolonged exposure to cigarette smoke induces parenchymal destruction and precipitates irreversible damage to the lungs [54], making quercetin an excellent candidate for antioxidant supplementation in pulmonary diseases.

Studies have shown that the cigarette smoke exposure (CS) method is a more suitable model of COPD for emphysema research due to its simple operation; however, research groups have associated more than one stimulus (CS + lipopolysaccharides/LPS) aimed to address more clinical manifestations, and observed that emphysema is induced in mice after 1 month of exposure to CS or CS + LPS, while airway remodeling was induced after 2 months of exposure to CS + LPS; the last one observed being greater collagen deposition and exhibited a more severe airflow limitation than mice in the CS group, suggesting that airflow limitation may be mainly induced by airway remodeling [55].

Overall, several major factors restrict the interpretation of data from animal models to human disease, which makes some limitations evident when using experimental models of COPD due to complex disease pathophysiology. It is important to highlight that when using a mouse model, the replication of severe COPD is not possible; there are other disadvantages as well, including low number of submucosal glands, monopodial airways branching, obligate nose-breather and pulmonary parameter measurement difficulty [56]. However, there are several strengths such as genetic heterogeneity, diverse responses to lung injury, low comparative costs and abundance of species-specific reagents [56]. This makes the animal model of cigarette smoke-induced COPD the best one, thus producing the inflammatory and pathogenic mechanisms of the disease as well as an appropriate model to study the process of emphysema [23,56], thus making it a primary testing methodology for drug therapies and representing a critical approach in deciding possible new drugs to treat COPD [57].

## 5. Conclusions

To our knowledge, this is the first evidence that quercetin not only protects against oxidative stress and lung inflammation, but also protects against pulmonary damage, improvement of antioxidants and pulmonary function in the COPD murine model. In conclusion, quercetin showed potent antioxidant and anti-inflammatory properties in the COPD murine model, established after 60 days of cigarette smoke exposure. Thus, these results suggest that quercetin might be an important agent in preventing pulmonary emphysema induced by cigarette smoke.

## Figures and Tables

**Table 1:** Composition of the experimental diet (g/1000 g of diet).

Nutrients (g)	Standard Diet
Carbohydrates	657
Protein	193
Fat	80
Fiber	10
Mineral mix ^a^	50
Vitamin mix ^a^	10
Energy density, kcal	4220

^a^ Vitamins and minerals mix following the Ain-93 M recommendation for rodents.

**Table 2:** Evaluation of lung function parameters in mice.

	CG	VG	QG	CSG	QCSG
RR (breaths/min)	163.0 ± 6.23	165.0 ± 13.97	162.8± 9.87	181.8 ± 1.94 ^a,b,c^	163.7± 2.58 ^d^
VT (mL)	0.302 ± 0.04	0.339 ± 0.03	0.340 ± 0.09	0.321 ± 0.04	0.341 ± 0.04
MV (mL/min)	49.38 ± 7.94	55.07 ± 8.29	55.07 ± 13.06	58.31 ± 6.91	55.65 ± 6.97

CG: control group; VG: vehicle group; QG: quercetin group; CSG: cigarette smoke group; QCSG: quercetin and cigarette smoke group; RR: respiratory rate; VT: tidal volume; MV: minute ventilation. The letter ^a^ represents a statistical difference between groups compared to CG. The letter ^b^ represents a statistical difference between groups compared to VG. The letter ^c^ represents a statistical difference between groups compared to QG. The letter ^d^ represents a statistical difference between groups compared to CSG. Data are expressed as mean ± standard deviation and statistical analyses were performed using the analysis of variance (one-way ANOVA) followed by Tukey’s post-test, *n* = 5–7 animals per group (*p* < 0.05).

**Table 3:** Hematological parameters of experimental groups.

	CG	VG	QG	CSG	QCSG
Erythrocytes (×10^6^/mm^3^)	7.99 ± 0.28	7.45 ± 0.29	7.53 ± 0.55	12.55 ± 1.28 ^a,b,c^	11.98 ± 0.95 ^a,b,c^
Hemoglobin (g/dL)	13.26 ± 0.56	11.91 ± 0.29	12.89 ± 0.56	19.09 ± 1.73 ^a,b,c^	19.16 ± 2.07 ^a,b,c^
Hematocrit (%)	36.61 ± 0.93	33.33 ± 0.42	34.64 ± 1.72	51.47 ± 5.90 ^a,b,c^	44.86 ± 3.81 ^a,b,c,d^

CG: control group; VG: vehicle group; QG: quercetin group; CSG: cigarette smoke group; QCSG: quercetin and cigarette smoke group. The letter ^a^ represents a statistical difference between groups compared to CG. The letter ^b^ represents a statistical difference between groups compared to VG. The letter ^c^ represents a statistical difference between groups compared to QG. The letter ^d^ represents a statistical difference between groups compared to CSG. Data are expressed as mean ± standard deviation and statistical analyses were performed using the analysis of variance (one-way ANOVA) followed by Tukey’s post-test, *n* = 5–7 animals per group (*p* < 0.05).

**Table 4:** Analysis of the cell influx to the bronchoalveolar lavage fluid.

	CG	VG	QG	CSG	QCSG
Total leucocytes (×10^3^/mL)	4.87 ± 1.02	4.12 ± 0.52	4.17 ± 0.70	25.17± 3.48 ^a,b,c^	13.13 ± 2.29 ^a,b,c,d^
Macrophages (×10^3^/mL)	4.84 ± 1.00	4.10 ± 0.52	4.15 ± 0.71	11.10 ± 2.13 ^a,b,c^	8.02 ± 1.47 ^a,b,c,d^
Lymphocytes (×10^3^/mL)	0.015 (0.00 ± 0.052)	0.00 (0.00 ± 0.052)	0.00 (0.00 ± 0.040)	1.36 (0.86 ± 2.12) ^a,b,c^	0.90 (0.69 ± 1.06) ^c^
Neutrophils (×10^3^/mL)	0.00 (0.00 ± 0.00)	0.00 (0.00 ± 0.00)	0.00 (0.00 ± 0.00)	12.33 (8.84 ± 16.62) ^a,b,c^	4.19 (3.53 ± 5.30)

CG: control group; VG: vehicle group; QG: quercetin group; CSG: cigarette smoke group; QCSG: quercetin and cigarette smoke group. The letter ^a^ represents a statistical difference between groups compared to CG. The letter ^b^ represents a statistical difference between groups compared to VG. The letter ^c^ represents a statistical difference between groups compared to QG. The letter ^d^ represents a statistical difference between groups compared to CSG. For total leucocytes and macrophages, the data are expressed as mean ± standard deviation and statistical analyses were performed using the analysis of variance (one-way ANOVA) followed by Tukey’s post-test, *n* = 6 animals per group (*p* < 0.05). For lymphocytes and neutrophils, the data are expressed in median, interquartile range (25% and 75%) values and were analyzed by Kruskal-Wallis followed by the Dunn’s posttest, *n* = 6 animals per group (*p* < 0.05).

**Table 5:** Inflammatory markers in lung parenchyma.

	CG	VG	QG	CSG	QCSG
IL-10 (pg/mL)	340.8 ± 136.81	287.4 ± 105.33	374.4 ± 92.11	631.6 ± 224.83 ^a,b,c^	370.6 ± 59.20 ^d^
IL-13 (pg/mL)	95.6 ± 23.46	104.4 ± 44.9	91.8 ± 30.21	240.4 ± 108.8 ^a,b,c^	135.6 ± 33,69 ^d^
IL-17 (pg/mL)	109.8 ± 27.09	159.6 ± 26.17	133.4 ± 13.58	264.2 ± 89.39 ^a,b,c^	385.8 ± 173.8 ^a,b^
IL-22 (pg/mL)	598.2 ± 166.15	649.8 ± 152.25	666.2 ± 257.44	918.8 ± 140.27 ^a^	547.2 ± 44.64 ^d^

CG: control group; VG: vehicle group; QG: quercetin group; CSG: cigarette smoke group; QCSG: quercetin and cigarette smoke group. IL-10: interleukin 10; IL-13: interleukin 13; IL-17: interleukin 17; IL-22: interleukin 22. The letter ^a^ represents a statistical difference between groups compared to CG. The letter ^b^ represents a statistical difference between groups compared to VG. The letter ^c^ represents a statistical difference between groups compared to QG. The letter ^d^ represents a statistical difference between groups compared to CSG. Data are expressed as mean ± standard deviation and statistical analyses were performed using the analysis of variance (one-way ANOVA) followed by Tukey’s post-test, *n* = 5–7 animals per group (*p* < 0.05).

**Table 6:** Biomarkers of oxidative stress in lung parenchyma of animals.

	CG	VG	QG	CSG	QCSG
SOD (U/mg protein)	30.54 ± 4.72	28.03 ± 8.79	27.26 ± 10.04	18.28 ± 4.94 ^a^	32.57 ± 3.69 ^d^
CAT (U/mg protein)	0.77 ± 0.29	0.68 ± 0.22	0.67 ± 0.30	0.32 ± 0.13 ^a^	0.77 ± 0.23 ^d^
GSH/GSSG ratio	2.94 (2.68–3.84)	2.61 (2.51–2.94)	2.74 (2.53–2.94)	1.75 (1.39–2.04) ^a,c^	1.90 (1.80–1.92) ^a^
MPO (U/mg protein)	0.21 ± 0.05	0.23 ± 0.04	0.23 ± 0.03	0.56 ± 0.29 ^a,b,c^	0.20 ± 0.03 ^d^
TBARS (nmol/mg protein)	0.76 ± 0.11	0.94 ± 0.30	0.78 ± 0.17	1.68 ± 0.68 ^a,b,c^	1.04 ± 0.28 ^d^
Protein carbonyl (nmol/mg protein)	6.36 ± 0.55	6.98 ± 2.71	7.74 ± 1.32	14.98 ± 2.10 ^a,b,c^	7.38 ± 1.65 ^d^

CG: control group; VG: vehicle group; QG: quercetin group; CSG: cigarette smoke group; QCSG: quercetin and cigarette smoke group. SOD: superoxide dismutase; CAT: catalase; GSH: glutathione sulfide; GSSG: oxidized glutathione; MPO: myeloperoxidase; TBARS: thiobarbituric acid reactive substances. The letter ^a^ represents a statistical difference between groups compared to CG. The letter ^b^ represents a statistical difference between groups compared to VG. The letter ^c^ represents a statistical difference between groups compared to QG. The letter ^d^ represents a statistical difference between groups compared to CSG. For SOD, CAT, TBARS and protein carbonyl the data are expressed as mean ± standard deviation and statistical analyses were performed using the analysis of variance (one-way ANOVA) followed by Tukey’s post-test, *n* = 5–7 animals per group (*p* < 0.05). For GSH/GSSG ratio data are expressed in median, interquartile range (25% and 75%) value and were analyzed by Kruskal-Wallis followed by the Dunn’s posttest, *n* = 5–7 animals per group (*p* < 0.05).

**Table 7:** Stereological and morphometric analyses of the lung tissue.

	CG	VG	QG	CSG	QCSG
Vv [a] (%)	60.00 (57.50–60.94)	55.31 (53.75–59.38)	57.50 (56.25–60.94)	66.25 (64.69–70.31) ^a,b,c^	55.00 (53.13–58.44) ^d^
Vv [sa] (%)	41.56 (39.38–44.06)	45.31 (44.38–47.19)	42.81 (41.56–44.69)	33.75 (29.69–35.31) ^a,b,c^	46.56 (41.88–48.44) ^d^
Vv [col] (%)	25.16 (23.20–30.47)	25.47 (24.53–29.69)	29.38 (21.25–34.53)	7.97 (6.50–9.30) ^a,b,c^	23.60 (19.69–24.69)
Vv [ela] (%)	18.13 (16.02–20.16)	17.50 (16.17–18.60)	16.10 (14.69–20.39)	12.19 (11.72–12.74) ^a,b,c^	15.94 (13.83–16.33)
Lm (µm)	127.6 ± 9.98	140.7 ± 9.72	121.2 ± 8.47	257.9 ± 69.61 ^a,b,c^	140.1 ± 6.88 ^d^

CG: control group; VG: vehicle group; QG: quercetin group; CSG: cigarette smoke group; QCSG: quercetin and cigarette smoke group. Vv [a]: volume densities of alveolar air space; Vv [sa]: volume densities of alveolar septa; Vv [col]: volume density of collagen fibers; Vv [ela]: volume density of elastic fibers. The letter ^a^ represents a statistical difference between groups compared to CG. The letter ^b^ represents a statistical difference between groups compared to VG. The letter ^c^ represents a statistical difference between groups compared to QG. The letter ^d^ represents a statistical difference between groups compared to CSG. For Lm, the data are expressed as mean ± standard deviation and statistical analyses were performed using the analysis of variance (one-way ANOVA) followed by Tukey’s post-test, *n* = 5–7 animals per group (*p* < 0.05). For Vv [a], Vv [sa], Vv [col] and Vv [ela], the data are expressed in median, interquartile range (25% and 75%) value and were analyzed by Kruskal-Wallis followed by the Dunn’s posttest, *n* = 5–7 animals per group (*p* < 0.05).

## Data Availability

The data presented in this study are available in this manuscript.
